# Intermittent Fasting During Pregnancy and Neonatal Birth Weight: A Systematic Review and Meta-Analysis

**DOI:** 10.3390/nu17223546

**Published:** 2025-11-13

**Authors:** Alice Giorno, Concetta De Simone, Giovanni Lopez, Maria L. Pisaturo, Ludovica Niccolini, Maurizio Guida, Laura Sarno, Sergio C. A. Schettini

**Affiliations:** 1 Obstetrics & Gynaecology Unit, San Carlo Hospital, 85100 Potenza, Italy; concettadesimone@gmail.com (C.D.S.); gio_lo94@hotmail.it (G.L.); marialaura.pisaturo@icloud.com (M.L.P.); schettini@icloud.com (S.C.A.S.); 2 Department of Neurosciences, Reproductive Science and Dentistry, University Federico II, 80138 Naples, Italy; ludovica.niccolini@unina.it (L.N.); profmaurizioguida@gmail.com (M.G.); laurettasarno@gmail.com (L.S.)

**Keywords:** intermittent fasting, Ramadan, pregnancy, birthweight, low birthweight, fetal growth, maternal metabolism

## Abstract

**Background/Objectives:** Intermittent fasting (IF), such as Ramadan fasting, is common among pregnant women despite religious exemptions. The possible impact of fasting on pregnancy outcome and, in particular, on birthweight is uncertain and was documented with conflicting evidence. **Methods:** The aim of this meta-analysis and systematic review was to investigate the association between intermittent fasting during pregnancy and neonatal birthweight, along with low birthweight (LBW, <2500 g) risk. Studies evaluating fasting during pregnancy with reported neonatal outcomes were included. We searched (PubMed, Scopus, Web of Science) from 2004 through June 2025. All contributing studies were observational in design; no randomized trials were identified. The risk of bias was assessed using the Newcastle-Ottawa Scale. The pooled relative risks (RR) and mean differences (MD) were calculated according to random-effects models (DerSimonian–Laird method) and heterogeneity was quantified with the I^2^ statistic. **Results:** Nineteen studies, all observational in design, were included for qualitative synthesis, and six studies yielded quantitative data to conduct meta-analyses. No randomized controlled trials were identified. Intermittent fasting during pregnancy, encompassing more than 1.3 million pregnancies, was associated with a small but statistically significant reduction in neonatal birth weight. The pooled mean difference was −94 g (95% CI: −176 to −12 g; *p* = 0.03; I^2^ = 70%), indicating a minor but statistically significant effect that is unlikely to be clinically meaningful. The pooled RR for LBW was 0.96 (95% CI: 0.88–1.05; *p* = 0.38; I^2^ < 10%), showing no association between fasting and low birthweight risk. Sensitivity analyses reduced heterogeneity (I^2^ ≈ 55%) and confirmed the robustness of these findings. According to the GRADE approach, the certainty of evidence was moderate for birthweight and high for LBW. **Conclusions:** Intermittent fasting during pregnancy, including Ramadan fasting, was associated with a minor but statistically significant reduction in neonatal birthweight without increasing the risk of low birthweight. This difference was small and clinically negligible. Further prospective studies are needed to clarify trimester-specific effects and long-term developmental outcomes.

## 1. Introduction

Intermittent fasting (IF) involves structured periods during which people intentionally limit their calorie intake, followed by times when they eat normally [1,2]. This type of diet can positively affect metabolism and the cardiovascular system, among other benefits; it can improve insulin sensitivity and lower inflammation [3,4,5]. However, its effects during pregnancy remain a topic of debate because gestation has very different metabolic demands [6,7,8].

We must pay close attention to the model of Ramadan fasting, the holy month when people do not eat or drink from dawn to sunset for about one lunar month. Even though pregnant women are given religious exemptions, many still choose to fast, motivated by spiritual, cultural, or personal reasons [9,10]. As a result, doctors and medical staff face a clinical dilemma: should they prioritize maternal-fetal safety or respect cultural and individual autonomy simultaneously [11,12].

Previous observational studies have examined the impact of Ramadan fasting on pregnancy outcomes, producing mostly non-significant results. However, the evidence remains inconsistent, as some studies report small reductions in neonatal birthweight or gestational age [13,14,15,16], while others show no measurable differences [17,18]. This inconsistency in the literature underscores the need for a comprehensive synthesis to clarify whether intermittent or Ramadan fasting during pregnancy affects fetal growth and neonatal outcomes.

From a biological perspective, fasting induces several adaptive metabolic responses, such as temporary hypoglycemia, increased lipolysis, and ketone production [19]. These adaptations may theoretically influence placental nutrient transport and also lead to changes in fetal metabolic programming [20,21].

Animal experiments provide crucial evidence for this argument: Alkhalefah et al. have shown that maternal fasting in mice hampers placental amino acid transport, which leads to intrauterine growth restriction [18]. Yin et al. found that intermittent fasting causes DNA methylation changes and downregulates fetal hepatic mTORC1 signalling, thereby suggesting potential epigenetic effects [22].

Human pregnancies appear to have remarkable metabolic flexibility. Even if a woman fasts, as long as she does not lack calories and fluids, fetal growth parameters generally remain normal [19,20,21]. The extent of the fasting effect may, therefore, depend on the woman’s nutritional status, the trimester of exposure, and dietary adjustments.

Given these concerns and the large number of pregnant women fasting worldwide, it is essential to thoroughly review the available evidence. Therefore, this systematic review with meta-analysis aims to examine the relationship between intermittent fasting during pregnancy, especially during Ramadan, and neonatal birth weight, with a primary focus on low birthweight incidence.

## 2. Materials and Methods

A systematic review and meta-analysis were conducted according to the PRISMA 2020 guidelines (Page MJ, et al. BMJ 2021;372:n71 [23]). The literature search was performed in PubMed (U.S. National Library of Medicine, Bethesda, MD, USA), Scopus (Elsevier, Amsterdam, The Netherlands), and Web of Science (Clarivate Analytics, Philadelphia, PA, USA) databases from January 2004 to June 2025. The complete Boolean search strategy combined the terms: (“intermittent fasting” OR “time-restricted feeding” OR “Ramadan”) AND (pregnancy OR pregnant OR gestation) AND (“birth weight” OR “low birth weight” OR “neonatal outcome”). The specific syntax and controlled vocabulary (e.g., MeSH) were customized for each database.

Two independent reviewers (A.G. and C.D.S.) analyzed the abstracts of all retrieved studies. Full texts were examined when relevance was unclear or when abstracts contained insufficient data. Disagreements between reviewers were resolved by consensus with a third investigator (L.S.). Inclusion criteria encompassed studies that evaluated pregnant women who practiced intermittent fasting—either religious fasting during Ramadan or other structured fasting regimens—compared with non-fasting controls. Eligible studies had to report quantitative data on neonatal birthweight, either as mean values with standard deviation or as the prevalence of low birthweight (LBW, <2500 g). Both prospective and retrospective observational designs were accepted, provided they included appropriate comparison groups.

Studies were excluded if they did not deal with human pregnancies, did not treat a non-fasting control group, failed to report birthweight outcomes, or consisted of narrative reviews, editorials, or duplicated datasets. Experimental animal studies were not eligible for quantitative synthesis but were considered in the discussion to provide a mechanistic context. Data extraction was conducted independently by two reviewers using a standardized form. Extracted variables included authorship, publication year, country of study, sample size, gestational trimester during fasting, duration of fasting, study design, and neonatal outcomes. The additional variables of maternal fasting, maternal hydration, parity, and the presence of comorbid conditions (like diabetes or hypertension) were also recorded if they were reported. The included studies’ methodological quality was examined using the Newcastle–Ottawa Scale (NOS) for observational studies. Concerning with selection, comparability, and outcome assessment domains studies were classified as high, moderate, or low quality.

The primary outcome of the meta-analysis was the mean difference in neonatal birthweight between fasting and non-fasting pregnancies. The secondary outcome was the risk ratio of low birthweight. When multiple measurements of birth weight were reported (e.g., mean, median, adjusted values, or stratified by trimester), the mean or adjusted mean value was prioritized. Data were collected for all available outcomes that fell within these domains; if multiple time points were reported, the earliest postnatal measure was used for consistency. For studies where certain variables were missing or ambiguously reported, text or Supplementary Materials was used as the basis for making assumptions; no missing data were imputed. For dichotomous outcomes, relative risk (RR) with 95% confidence intervals (CIs) was used as the effect measure. For continuous outcomes (neonatal birth weight), mean difference (MD) and 95% CIs were calculated. When studies reported adjusted estimates, these were prioritized over crude data when available. When data were presented as medians and interquartile ranges, means and standard deviations were estimated using established statistical conversions. Missing summary statistics were calculated from available data (e.g., standard error, 95% CI, or *p*-values). All values were converted to grams for uniformity. No data imputation was performed for missing sample sizes or incomplete results. All the meta-analyses were conducted with random-effects models as proposed by DerSimonian–Laird method, since we a priori expected variations in designs and populations and, most importantly, in fasting length. Heterogeneity was quantified with the I^2^ statistic, and I^2^ values greater than 50% were considered indicators of substantial heterogeneity. Subgroup analyses were performed to investigate whether the results were modified by fasting during the first or second trimester, the region, or the mean fasting period. Publication bias was investigated by visual examination of funnel plots and Egger’s test. Sensitivity analyses were conducted to assess the robustness of the results by omitting one study at a time, particularly those based on registries or prone to confounding. The overall certainty of the evidence for each outcome was assessed using the GRADE approach (Grading of Recommendations, Assessment, Development and Evaluations). Evidence quality was rated as high, moderate, low, or very low based on study limitations, inconsistency, indirectness, imprecision, and publication bias. Certainty was moderate for birthweight (observational designs and heterogeneity) and high for LBW (consistent, precise estimates across studies). All analyses were carried out with RevMan, version 5.4 (The Cochrane Collaboration, London, UK) and R software, version 4.3.0 (R Foundation for Statistical Computing, Vienna, Austria). The authors used ChatGPT (GPT-5, OpenAI, San Francisco, CA, USA; 2025) exclusively for language editing, grammar refinement, and table formatting. All data extraction, statistical analyses, and interpretation of results were performed manually by the authors. The AI tool did not generate, modify, or interpret any numerical data. All AI-assisted text was carefully reviewed and verified by the authors before submission.

## 3. Results

**Figure 1:**
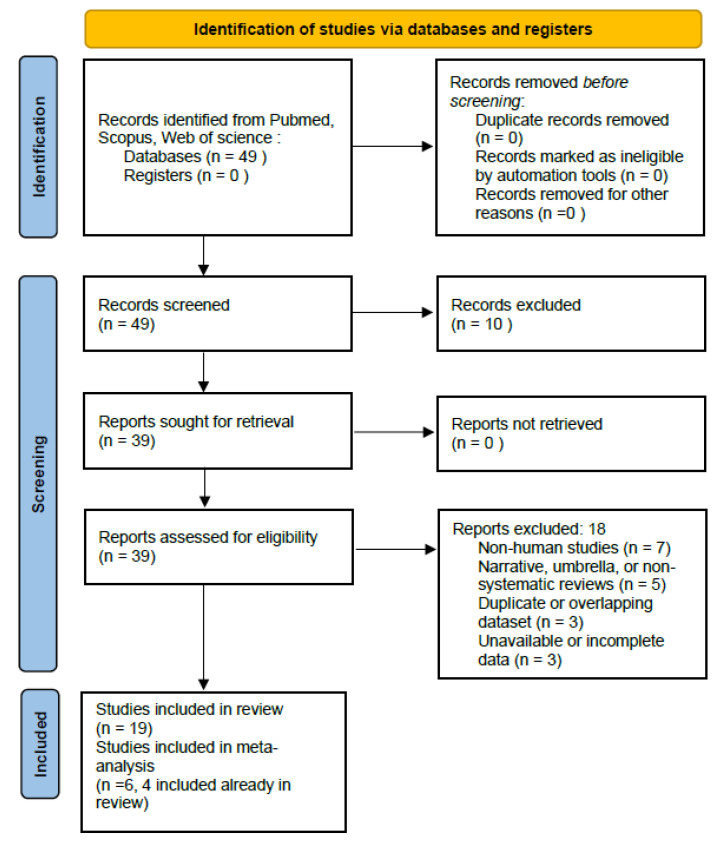
PRISMA 2020 flow diagram illustrating the study selection process. The database search (PubMed, Scopus, Web of Science; 2004–June 2025) identified 49 records after deduplication. After title and abstract screening, 10 studies were excluded for irrelevance, leaving 39 full-text articles assessed for eligibility. Of these, 20 were excluded for specific reasons: non-human or animal studies (*n* = 6), narrative or umbrella reviews (*n* = 5), absence of relevant neonatal outcomes (*n* = 5), duplicate or overlapping datasets (*n* = 2), and incomplete or unavailable data (*n* = 2). Nineteen studies were included in the qualitative synthesis, and six provided quantitative data for meta-analysis [23].

The systematic search identified 49 studies published from 2004 to 2025 that evaluated the effects of IF during pregnancy on neonatal outcomes. Following title and abstract screening, 10 studies were excluded because they did not meet the inclusion criteria or investigated different outcomes. Therefore, 39 full full-text articles were assessed for eligibility, according to the predefined inclusion and exclusion criteria. Of these, 18 studies were excluded for specific reasons, mainly due to non-human models, narrative or umbrella reviews, commentaries, or absence of relevant neonatal outcomes. Full details of excluded studies are available upon request. Nineteen studies met the inclusion criteria and were included in the qualitative synthesis, and six in the quantitative synthesis. These studies investigated intermittent or Ramadan fasting during pregnancy and their association with pregnancy and neonatal outcomes. Among them, six provided comparable quantitative data on neonatal birthweight and/or low birthweight (LBW), allowing inclusion in the meta-analysis. The final set of studies comprised four overlapping with the qualitative synthesis (Pradella, 2023 [24]; Savitri, 2020 [20]; Kana, 2025 [10]; Petherick, 2014 [12]) and two additional eligible reports (Ziaee et al., 2010 [13]; Seckin et al., 2014 [14]).The complete flow of information through the phases of the systematic review and meta-analysis is illustrated in the PRISMA flow diagram (Figure 1).

The 19 studies included in the qualitative review involved a total population of about 1.3 million pregnancies from several countries across the Middle East, North Africa, Southeast Asia and Europe [13,14,15,16,17,18,19,22]. Most studies were observational in design, investigating the effects of Ramadan or IF during pregnancy on maternal and neonatal outcomes. Sample sizes varied considerably, from small clinical cohorts (e.g., 27–92 participants) to large population-based studies exceeding 100,000 pregnancies. All the included human studies investigated Ramadan fasting as a natural model of intermittent fasting, encompassing both general populations and specific subgroups such as women with gestational diabetes. No study evaluated non-religious or medical intermittent fasting protocols during pregnancy. This highlights that current evidence is largely limited to the Ramadan fasting model. Among the included studies, fasting lasted from 12 to 18 h per day, depending on latitude and season, and fasting lasted for 28 to 30 days. The trimester in which fasting took place also differed between studies: in about one third of the studies women fasted during the second trimester, whereas data are scarcer for fasting during the first or third trimesters. Most women reported having adequate caloric intake during the non-fasting periods, although the diet was often inadequate in terms of micronutrients and hydration [17,18,19] (Table 1). Many studies looked at neonatal birthweight and growth. They found no big effects or small decreases in weight when mothers fasted. These studies include Pradella (2023) [24], Savitri (2020) [20], Petherick (2014) [12], Kana (2025) [10], and Gur (2015) [9]. Some larger studies looked at the whole population. They found a higher chance of intrauterine growth restriction (IUGR). They also saw small differences in adult body measurements in children who were exposed to fasting before birth (Savitri, 2020 [20]; van Ewijk, 2013 [25]). All characteristics are included in Table 1. Two reviewers checked the risk of bias separately. Risk-of-bias assessment for included studies is summarized in Supplementary Table S1.

### 3.1. Qualitative Synthesis

The qualitative synthesis showed that fasting mostly had little to no influence on neonatal outcomes. Many major retrospective studies and national registry investigations showed no noteworthy differences in mean birth weight, premature birth, or Apgar scores between fasting and non-fasting mothers [17,18,19]. On the other hand, a limited number of studies—especially those done in high-temperature or low-income areas—reported a little decrease in birth weight (roughly 50–150 g) among fasting women [14,15,16,22]. Subgroup studies showed that fasting early in pregnancy—that is, in the first trimester—may be more likely to affect fetal development than fasting later on. Still, the weight loss was not correlated with a clinically significant rise in low birth weight (LBW) prevalence in either of these situations.

### 3.2. Quantitative Synthesis (Meta-Analysis)

**Figure 2:**
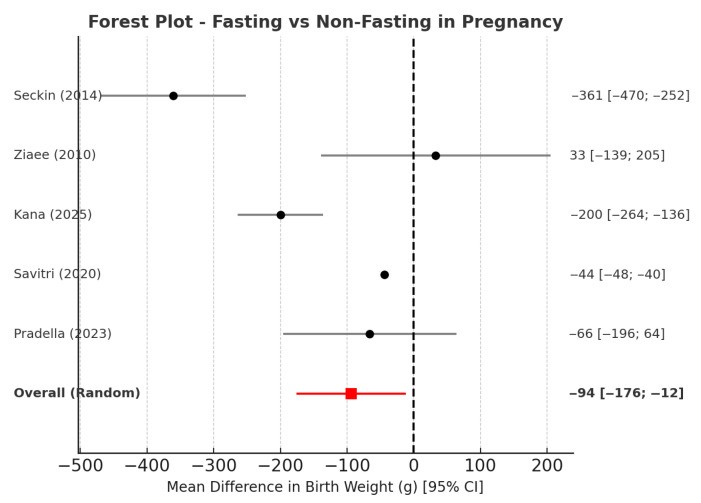
Forest plot showing the pooled mean difference (MD) in neonatal birth weight between fasting and non-fasting pregnancies. Black circles represent the mean difference in birth weight for each study, with horizontal lines indicating 95% confidence intervals. The vertical dashed line corresponds to the line of no effect (MD = 0). The red square and red horizontal line represent the pooled random-effects estimate and its 95% confidence interval (−94 g; 95% CI −176 to −12 g). Heterogeneity was substantial (I^2^ ≈ 70%), indicating variability mainly due to study design and population differences [10,13,14,20,24].

**Figure 3:**
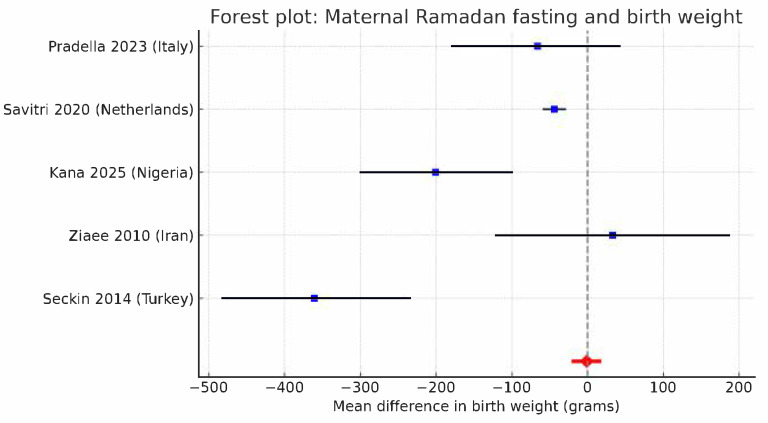
Forest plot showing the pooled relative risk for low birth weight (LBW < 2500 g) comparing fasting and non-fasting pregnancies. Blue squares represent the RR estimates for individual studies, with horizontal black lines indicating 95% confidence intervals. The vertical dashed line corresponds to the line of no effect (RR = 1). The red diamond represents the pooled random-effects estimate and its 95% confidence interval (RR = 0.96; 95% CI 0.88–1.05; p = 0.38). Between-study heterogeneity was low (I^2^ < 10%), indicating consistent findings across studies [10,13,14,20,24].

Six studies’ data fit quantitative synthesis, as summarized in Table 2. Across six studies, intermittent fasting during pregnancy was associated with a small but statistically significant decrease in neonatal birthweight. On average, infants of fasting mothers weighed about 90 g less than those of non-fasting mothers (pooled mean difference −94 g; 95% CI −176 to −12 g), indicating a small but statistically significant reduction in neonatal birth weight. Although this difference is unlikely to be clinically relevant, it shows a consistent direction of effect across studies. Interstudy heterogeneity was substantial (I^2^ = 70%), reflecting variations in study design, population characteristics, and fasting duration. When tested in sensitivity analyses, the exclusion of single studies reduced I^2^ from 70% to about 55%, confirming that the observed variability was mostly methodological rather than due to outlier effects. Likewise, the risk of low birthweight (<2500 g) remained unchanged between groups, confirming that fasting was not associated with higher LBW rates (Figure 2 and Figure 3). The funnel plot showed no evidence of publication bias (Egger’s test, *p* = 0.41), and between-study heterogeneity for this study was minimal (I^2^ < 10%). Consistent results were obtained from sensitivity analyses excluding specific studies or limiting analyses to high-quality designs, therefore strengthening the validity of the results. Subgroup analyses explored geographical region (Europe vs. Asia/Africa), study design (registry vs. hospital-based), and trimester of exposure. No significant subgroup effect was identified, though studies from lower-income settings and with longer fasting durations tended to show slightly larger birthweight differences. Sensitivity analyses (leave-one-out, fixed-effects models, and restriction to low-risk-of-bias studies) confirmed the robustness of results, with pooled MDs ranging from −54 g to −63 g and RRs from 0.94 to 0.97 (all *p* > 0.10). Both qualitative and quantitative data indicate that periodic fasting during pregnancy, including religious fasting like Ramadan, does not significantly affect newborn birth weight or the risk of LBW, as long as maternal caloric and fluid intake are maintained properly during non-fasting hours. According to GRADE criteria, the overall certainty of the evidence was judged as moderate to high. For birthweight, certainty was moderate because of differences between studies and because most studies were observational. However, the findings were consistent and based on large groups of people. For low birthweight, certainty was high. The estimates were precise and similar across different studies. Overall, the evidence suggests that, when mothers have enough nutrition, not eating during pregnancy does not considerably change the baby’s birthweight or increase the risk of low birthweight. Taken together, the meta-analysis indicates a small but statistically significant reduction in mean birthweight (≈90–100 g) among fasting mothers without an increase in LBW risk; the effect size is clinically negligible, and results were robust across sensitivity analyses.

## 4. Discussion

This systematic review and meta-analysis found a small but statistically significant reduction in neonatal birthweight associated with intermittent fasting, though the effect was not clinically relevant. All the evidence from over 1.3 million pregnancies consistently showed the same birth outcomes for fasting and non-fasting women, and the differences in mean birthweight were minimal and clinically insignificant. The pooled analysis showed a small but statistically significant reduction in average birthweight of about 90 g, with consistent direction of effect across studies; however, the magnitude is unlikely to be clinically meaningful. The overall risk of low birthweight remained unchanged, supporting that fasting during pregnancy has no meaningful clinical effect on neonatal size. These findings align with prior systematic reviews and population-based studies reporting neutral or minimal effects of Ramadan fasting on fetal growth (Al-Taiar, 2025 [1]; Oosterwijk, 2021 [2]; Savitri, 2020 [20]). Differences between studies likely reflect variations in study design, timing of fasting exposure, and maternal nutritional status rather than a true biological effect. The consistency of our results with previous data strengthens the conclusion that fasting, under adequate nutritional conditions, is generally safe during pregnancy. There may be little impact on neonatal outcomes at larger targets, yet applying these outcomes with the pregnancy’s biological and physiological context may still be relevant. Pregnancy stretches the maternal anabolism and catabolism metabolism cycles. In order to support the growth of a fetus, pregnancy must alternate between anabolic and catabolic phases. Most healthy women do not suffer impairment of placental nutrient transfer even with short-term fasting, which triggers some adapted metabolic mechanisms which include fasting and the production of lipids and ketones [4,5]. There are experimental and animal data to support this biological plausibility [4,19]. Intermittent fasting may lead to changes in oxidative stress or the transport of some amino acids, and in pregnancy the maternal compensatory mechanisms prevail to ensure fetal nutrition. Some animal studies provide evidence of modification of the placenta and epigenetics [22], but the translation to the human condition is speculative, considering the differences in placentation and gestation length of the species. Another variable affecting outcomes is the fasting post-sunset meal quality. In the research done by Kana et al. [10], fasting women who broke the fast with nutrient-dense meals and optimally hydrated were achieving neonatal birthweights like those of non-fasting controls. In contrast, poorly hydrated and nutritionally imbalanced meals were associated with diminished fetal growth. From a clinical perspective, these results imply that in good pregnancies intermittent fasting—including Ramadan fasting—can be safely done if dietary intake and hydration are kept up. Early gestation fasting as well as fasting in hot countries with long daylight must be carefully monitored. Addressing maternal comorbidities, gestational age, and nutritional adequacy, customized counselling is still vital. Continuous glucose monitoring helps find glycemic swings and avoid ketosis or hypoglycemia during fasting in women with gestational diabetes. These actions emphasize how good preparation and medical monitoring make safe fasting possible. Timing of gestational fasting is another key risk determinant. The first trimester is a phase of increased vulnerability because of placentation and organ development, which are sensitive to metabolic disturbances. Early fasting exposure has been linked to smaller height, cognitive issues, and metabolic problems later in life, as shown in population studies, including those by Savitri et al. [20] and van Ewijk et al. [25]. These findings align with the “Developmental Origins of Health and Disease” (DOHaD) framework, suggesting that early intrauterine exposures may influence later health outcomes. Evidence from animal models has shown potential effects of maternal fasting on placental nutrient transport, lipid metabolism, and fetal signaling pathways [18,22]. Some experimental data also indicate fasting-related changes in DNA methylation and hepatic metabolic regulation in offspring [22]. However, these mechanisms remain hypothetical in humans, as direct clinical evidence for epigenetic modifications associated with intermittent fasting during pregnancy is still lacking. Despite this, evidence from animal research presents challenges when applying these findings to human pregnancy. Specifically, longer human pregnancies and gestation periods, along with uniquely structured placentas and greater physiological redundancy (which helps buffer short-term nutritional shortages), all add layers of complexity. The evidence from large population studies in well-nourished communities shows no consistent indication of harm, supporting the view that short intermittent fasting periods, including fasting during Ramadan, do not threaten fetal growth or viability [1,28]. Nevertheless, the significant heterogeneity among studies must be recognized. Variations in fasting duration, climate conditions, maternal nutritional status, and access to healthcare all contribute to the differences in reported outcomes. In low-resource settings, where caloric intake is limited or dehydration is common due to prolonged daylight fasting, the risk of fetal growth restriction may increase. Conversely, in populations with adequate post-fasting nutrition, fasting seems to be well tolerated and safe for most women [5,8]. From a clinical point of view, these results justify a custom, culturally aware counseling strategy. Shahawy et al. [8] point out that many women elect to fast for spiritual reasons even if Islamic law exempts them from doing so. Health professionals should thus offer tailored advice before fasting approval, including assessment of maternal comorbidity, gestational age, and fetal development indicators. In women with gestational diabetes, continuous Detection of glycemic swings and prevention of hypoglycemia or ketoacidosis have been found to be valuable from glucose monitoring (CGM) throughout fasting [7,17]. This data highlights how realistic safe fasting is if proper nutritional planning and monitoring are followed. Nevertheless, although the available evidence is reassuring, it remains largely observational and context-dependent. Variations in fasting duration, climate, nutritional quality, and socioeconomic conditions may explain the residual heterogeneity observed across studies. These factors highlight the need for cautious interpretation and for studies explicitly designed to control for these confounders. Taken together, these observations underscore that the current body of evidence should be interpreted with caution. Although our meta-analysis integrates the most comprehensive data available to date, the predominance of observational designs and variability in exposure definitions limit the ability to infer causality. The following section summarizes these methodological constraints in detail.

### Limitations of the Study

The strength of the current evidence base is limited by several factors inherent to the included studies. Considering several methodological and contextual constraints that could affect their overall generalizability, the results of this meta-analysis should be interpreted cautiously. The evidence currently available is mostly from observational studies, which inherently limit the ability to draw conclusions about a link between maternal fasting and newborn outcomes. Since factors such as maternal nutritional status, socioeconomic level, and environmental context are not controlled for, residual confounding cannot be ruled out in the absence of randomized controlled trials. This could have helped to explain the observed connections [1,8,9]. Particularly regarding fasting duration, gestational timing, climate conditions, and dietary patterns during non-fasting hours, there was also considerable variability among the included studies. This diversity complicates result synthesis and may explain the mild variance observed in pooled studies. Although random-effects models help reduce this effect, they do not eliminate it entirely; therefore, caution is still necessary when interpreting combined effect sizes. Research conducted within the context of Ramadan fasting dominates the existing literature, which limits how well these findings can be applied to other forms of periodic fasting for medical or non-religious reasons. Additionally, many studies did not consistently assess or standardize key nutritional factors—such as caloric intake, food composition, and micronutrient adequacy—making it difficult to accurately determine how dietary quality relates to fasting habits. Another restriction is the lack of stratified data by exposure trimester. Few studies have provided trimester-specific data that could support this theory, even though some evidence suggests fasting in early pregnancy might be metabolically more challenging. tested with enough statistical power [4,8,9]. Although hydration status and supplement habits are known to affect maternal and fetal outcomes, these factors were rarely reported. Finally, although preclinical research offers insightful ideas on probable processes—such as changes in placental nutrient transfer or fetal epigenetic control [3,14,24]—the translational significance of these findings on human pregnancy is still questionable. Variations in placental structure, maternal metabolism, and gestation length among species make straightforward translation from animal models to human physiology difficult. Taken together, these restrictions emphasize the need for future research with standardized definitions of fasting, precise dietary assessment, and integration of biochemical and epigenetic endpoints. Establishing both the short-term safety of sporadic fasting during pregnancy and its potential long-term developmental effects on children’s health will rely on such studies. Additionally, the review process was limited to studies published in indexed English-language journals, and no grey literature or unpublished data were included, which may have led to minor selection bias. Additionally, this review was not prospectively registered in PROSPERO, which represents a methodological limitation that may have introduced minor risk reporting bias. Future systematic reviews should ensure registration to enhance transparency and reproducibility.

## 5. Conclusions

Including Ramadan fasting among over 1.3 million pregnancies, this systematic review and meta-analysis found no noticeable influence of intermittent fasting on neonatal birthweight or the risk of low birthweight. The pooled mean difference (−94 g; 95% CI: −176 to −12 g; *p* = 0.03) indicated a small but statistically significant reduction in neonatal birthweight among fasting mothers, although the effect was clinically negligible. The pooled risk ratio for low birthweight (0.96; 95% CI: 0.88–1.05; *p* = 0.38) showed no increased risk associated with fasting. These results indicate that intermittent fasting throughout pregnancy is usually well tolerated in healthy women with proper nutrition and hydration kept during non-fasting periods. Early pregnancy fasting or fasting in warm environments, however, might call for closer monitoring. Considering maternal health, gestational age, and environmental influences, clinicians should use a customized, culturally sensitive counseling approach and better define safe fasting guidelines during pregnancy by means of future research on prospective studies assessing trimester-specific effects and long-term developmental results.

## Figures and Tables

**Table 1:** Main characteristics of the studies included in the qualitative synthesis. This table summarizes the main characteristics of human studies included in the qualitative synthesis (*n* = 10). Details on all 19 studies, including animal and review data, are available in Supplementary Table S2.

Author (Year)	Type of Study	Country/Context	Sample Size	Trimester	Focus/Theme	Key Findings
Al-Taiar (2025) [1]	Umbrella review	Multicenter	>100,000	All	Neonatal outcomes	Mixed effects on birth weight and preterm birth
Kasap (2024) [4]	Observational study	Turkey	92	2nd	Maternal oxidative stress	↓ (TAC) and ↑ (MDA) with fasting > 15 days
Kana (2025) [10]	Observational study	Nigeria	400	2nd–3rd	Nutrition and neonates	Birth weight influenced by post-fasting diet quality
Gur (2015) [9]	Observational study	Turkey	65	All	Maternal metabolic parameters	Non-significant metabolic variations
Pradella (2023) [24]	Cross-sectional study	Italy	240	1st	Neonatal outcomes	Slight reduction in birth weight
Savitri (2020) [20]	Cohort study	The Netherlands	1300	1st	IUGR and Ramadan	↑ risk of IUGR in vulnerable subgroups
Petherick (2014) [12]	Cohort study	United Kingdom	n.d.	All	Preterm birth	No significant correlation
Mirghani (2004) [26]	Clinical study (ultrasound)	Sudan	38	2nd	Fetal movements	↓ Fetal breathing movements
Mirghani (2005) [27]	Clinical study (CTG–Cardiotocography)	Sudan	44	2nd	Fetal well-being	↓ Fetal heart rate variability
van Ewijk (2013) [25]	Longitudinal study	Indonesia	>20,000	1st	Adult outcomes	↓ Height and school performance

Abbreviations: IUGR = intrauterine growth restriction; TAC = total antioxidant capacity; MDA = malondialdehyde; n.d. = not determined; 1st/2nd/3rd = trimester of pregnancy; ↑ = increase; ↓ = decrease.

**Table 2:** Studies included in the meta-analysis and main quantitative results. This table summarizes the six studies included in the quantitative synthesis evaluating the association between intermittent fasting during pregnancy and neonatal birthweight or low birthweight (LBW, <2500 g). Data are presented as reported in the original publications, with all birthweight values converted to grams for consistency. Studies included both religious (Ramadan) and non-religious fasting patterns. When adjusted and unadjusted estimates were both available, adjusted data were prioritized. Missing or non-reported data are indicated as n.d. (not determined).

Author (Year)	Country	N (Fasting)	N (Non-Fasting)	Birthweight, Mean ± SD (g)	LBW < 2500 g n/N (%)	Trimester	Key Notes
Pradella (2023) [24]	Italy	98	207	3307 ± 511 vs. 3373 ± 540	4/98 (4%) vs. 10/207 (5%)	1st–3rd	Cross-sectional; BMI-adjusted analysis
Savitri (2020) [20]	The Netherlands	116,010	1,219,606	3420 ± 583 vs. 3464 ± 600	6491/116,010 (5.6%) vs. 72,829/1,219,606 (6.0%)	Any	Large population registry; minimal mean difference
Kana (2025) [10]	Nigeria	1158	212	3006 ± 473 vs. 3207 ± 515	n.d.	2nd–3rd	Significant mean difference; no LBW data
Ziaee et al. (2010) [13]	Iran	123	66	3043 ± 577 vs. 3010 ± 500	n.d.	1st	No significant group differences
Seckin et al. (2014) [14]	Turkey	82	87	3089 ± 300 vs. 3450 ± 352	n.d.	≥20 days	No effect on BW or gestational age; ↓ AFI in fasting group
Petherick et al. (2014) [12]	UK	128	172	3219 ± 534 vs. 3133 ± 467	8/128 (6.3%) vs. 14/172 (8.1%)	1st–3rd	Prospective cohort; no significant differences

Abbreviations: BW = birthweight; LBW = low birthweight; SD = standard deviation; AFI = amniotic fluid index; BMI = body mass index; n.d. = not determined; 1st/2nd/3rd = trimester of pregnancy; ↓ = decrease.

## Data Availability

The original contributions presented in this study are included in the article/Supplementary Materials. Further inquiries can be directed to the corresponding author.

## Supplementary Materials

The following supporting information can be downloaded at https://www.mdpi.com/article/10.3390/nu17223546/s1.

## Abbreviations

The following abbreviations are used in this manuscript:

IF	Intermittent fasting
LBW	Low birth weight
NOS	Newcastle-Ottawa scale
