# Supplementary material for: Intermittent Fasting During Pregnancy and Neonatal Birth Weight: A Systematic Review and Meta-Analysis
Source: Nutrients. 2025 Nov 13;17(22):3546. doi: 10.3390/nu17223546 (PMC12655342; doi:10.3390/nu17223546)
Supplement: Supplementary file 1 [file nutrients-17-03546-s001.zip › Figure S1. PRISMA_2020_flow_diagram_new_SRs_v1.pdf]

PRISMA 2020 flow diagram for new systematic reviews which included searches of databases and registers only

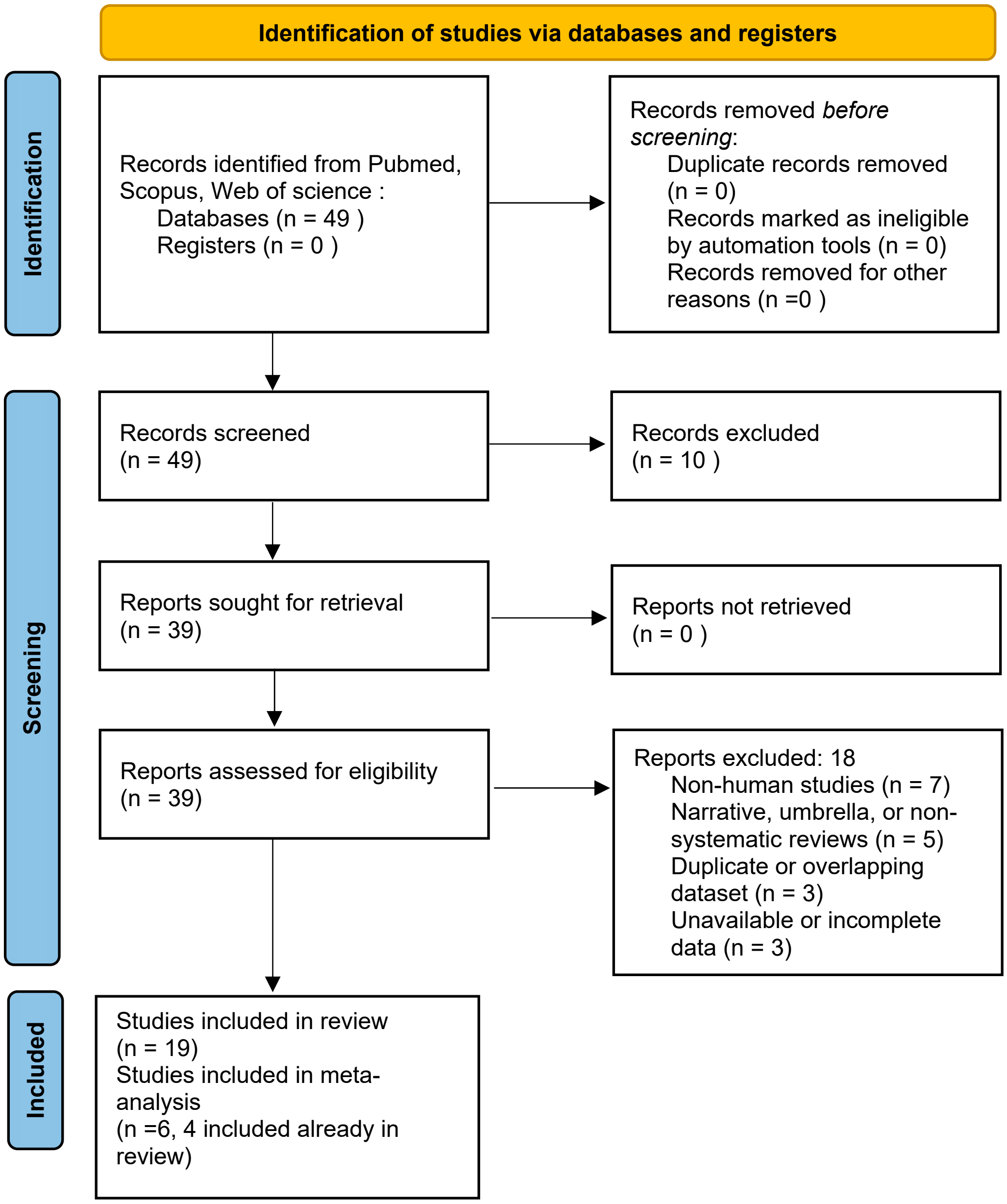

Figure S1. PRISMA 2020 flow diagram illustrating the study selection process.

The database search (PubMed, Scopus, Web of Science; 2004–June 2025) identified 49 records after deduplication. After title and abstract screening, 10 studies were excluded for irrelevance, leaving 39 full-text articles assessed for eligibility. Of these, 20 were excluded for specific reasons: non-human or animal studies (n = 6), narrative or umbrella reviews (n = 5), absence of relevant neonatal outcomes (n = 5), duplicate or overlapping datasets (n = 2), and incomplete or unavailable data (n = 2). Nineteen studies were included in the qualitative synthesis, and six provided quantitative data for meta-analysis

Source: Page MJ, et al. BMJ 2021;372:n71. doi: 10.1136/bmj.n71.

This work is licensed under CC BY 4.0. To view a copy of this license, visit https://creativecommons.org/licenses/by/4.0/
