# Supplementary material for: Intermittent Fasting During Pregnancy and Neonatal Birth Weight: A Systematic Review and Meta-Analysis
Source: Nutrients. 2025 Nov 13;17(22):3546. doi: 10.3390/nu17223546 (PMC12655342; doi:10.3390/nu17223546)
Supplement: Supplementary file 1 [file nutrients-17-03546-s001.zip › PRISMA Checklist.pdf]

PRISMA 2020 Main Checklist

Topic	No.	Item	Location where item is reported
**TITLE**			
**Title**	1	Intermittent Fasting During Pregnancy and Neonatal Birth Weight: A Systematic Review and Meta-Analysis	Page 1
**ABSTRACT**			
**Abstract**	2	Background/Objectives: Intermittent fasting (IF), such as Ramadan fasting, is common among pregnant women despite religious exemptions. The possible impact of fasting on pregnancy outcome and, in particular, on birthweight is uncertain and was documented with conflicting evidence (1-4). Methods: The aim of this me-ta-analysis and systematic review was to investigate the association between intermit-tent fasting during pregnancy and neonatal birthweight, along with low birthweight (LBW, <2500 g) risk. We searched (PubMed, Scopus, Web of Science) from 2004 through June 2025. The risk of bias was assessed using the Newcastle-Ottawa Scale. The pooled relative risks (RR) and mean differences (MD) were calculated according to random-effects models (DerSimonian–Laird method) and heterogeneity was quanti-fied with the I2 statistic. Results: Nineteen studies were included for qualitative syn-thesis, and five studies yielded quantitative data to conduct meta-analyses. Intermit-tent fasting during pregnancy, encompassing more than 1.3 million pregnancies, was not associated with a clinically significant decrease in neonatal birth weight. The pooled mean difference was −58 g (95% CI: −132 to +16 g; $p = 0.12$; $I^2 = 70\%$) and the pooled RR for LBW was 0.96 (95% CI: 0.88–1.05; $p = 0.38$; $I^2 < 10\%$). Subgroup and sen-sitivity analyses reaffirmed these results. Conclusions: Intermittent fasting during pregnancy, such as Ramadan fasting, doesn't seem to influence neonatal birth-weight or LBW risk significantly. Further prospective studies are required to assess the potential long-term effect and trimester-specific impact. The available evidence is lim-ited by the observational nature of included studies, variability in fasting duration and dietary reporting, and incomplete stratification by gestational age.	Page 1
**INTRODUCTION**			

Topic	No.	Item	Location where item is reported
**Rationale**	3	Intermittent fasting (IF) involves structured periods during which people intentionally limit their calorie intake, followed by times when they eat normally. This type of diet can positively affect metabolism and the cardiovascular system, among other benefits; it can improve insulin sensitivity and lower inflammation. However, its effects during pregnancy remain a topic of debate because gestation has very different metabolic demands.We must pay close attention to the model of Ramadan fasting, the holy month when people do not eat or drink from dawn to sunset for about one lunar month. Even though pregnant women are given religious exemptions, many still choose to fast, motivated by spiritual, cultural, or personal reasons. As a result, doctors and medical staff face a clinical dilemma: should they prioritize maternal-fetal safety or respect cultural and individual autonomy simultaneously.	Pag. 1-2
**Objectives**	4	this systematic review with meta-analysis aims to examine the relationship between intermittent fasting during pregnancy, especially during Ramadan, and neonatal birth weight, with a primary focus on low birthweight incidence.	Line 72-74
**METHODS**			
**Eligibility criteria**	5	Inclusion criteria encompassed studies that evaluated pregnant women who practiced intermittent fasting—either religious fasting during Ramadan or other structured fasting regimens—compared with non-fasting controls. Eligible studies had to report quantitative data on neonatal birthweight, either as mean values with standard devia-tion or as the prevalence of low birthweight (LBW, <2500 grams).Studies were excluded if they did not deal with human pregnancies, did not treat a non-fasting control group, failed to report birthweight outcomes, or consisted of narrative reviews, editorials, or duplicated datasets	Line 86-90 ; 97-99
**Information sources**	6	The literature search was performed in PubMed, Scopus, and Web of Science databases from January 2004 to June 2025.	Line 78-79
**Search strategy**	7	The search strategy included combinations of the following key terms: "intermittent fasting," "Ramadan," "pregnancy," "birth weight," "low birth weight," "neonatal outcomes," and "maternal fasting." Boolean operators and MeSH terms were tailored for each database to ensure maximum sensitivity.	Line 79-82

Topic	No.	Item	Location where item is reported
**Selection process**	8	Two independent reviewers (A.G. and C.D.S.) analyzed the abstracts of all re-trieved studies. Full texts were examined when relevance was unclear or when ab-stracts contained insufficient data. Disagreements between reviewers were resolved by consensus with a third investigator (L.S.).	Line 83-100
**Data collection process**	9	Data extraction was conducted independently by two reviewers using a standardized form. For each included article, information was extracted on study design, population characteristics, fasting exposure (type, duration, trimester), outcomes assessed, and key statistical results. When essential data were incomplete or unclear, attempts were made to contact the corresponding authors via email to obtain clarification or missing values. No automation tools or artificial intelligence software were employed in the data extraction process; all data were verified manually by the review team.	Line 99-105
**Data items**	10a	The primary outcome of interest was neonatal birth weight (in grams). The secondary outcome was the incidence of low birth weight (LBW), defined as <2500 g. When multiple measurements of birth weight were reported (e.g., mean, median, adjusted values, or stratified by trimester), the mean or adjusted mean value was prioritized. Data were collected for all available outcomes that fell within these domains; if multiple time points were reported, the earliest postnatal measure was used for consistency.	Line 106-112
	10b	Along with outcome data, we also collected the following study-level variables: authorship, year of publication, country, study design, sample size, age of the mother, gestational age at the time of fasting, duration and timing of fasting (trimester), and whether the fasting was religious (Ramadan) or secular. The additional variables of maternal fasting, maternal hydration, parity, and the presence of comorbid conditions (like diabetes or hypertension) were also recorded if they were reported. For studies where certain variables were missing or ambiguously reported, text or supplementary material was used as the basis for making assumptions; no missing data were imputed.	Line 98-102

Topic	No.	Item	Location where item is reported
**Study risk of bias assessment**	11	The risk of bias of the included studies was independently assessed by two reviewers (A.G. and C.D.) using the Newcastle–Ottawa Scale (NOS) for observational studies. The scale evaluates three domains: selection of participants, comparability of study groups, and ascertainment of outcomes. Each study was assigned a score from 0 to 9, with higher scores indicating lower risk of bias. Disagreements between reviewers were resolved by consensus or consultation with a third reviewer (L.S.). No automation tools or software were used for bias assessment; all evaluations were performed manually.	Line 103-105
**Effect measures**	12	For dichotomous outcomes, relative risk (RR) with 95% confidence intervals (CIs) was used as the effect measure. For continuous outcomes (neonatal birth weight), mean difference (MD) and 95% CIs were calculated. When studies reported adjusted estimates, these were prioritized over crude data when available.	Line 115-119
**Synthesis methods**	13a	Eligible studies had to report quantitative data on neonatal birthweight, either as mean values with standard deviation or as the prevalence of low birthweight (LBW, <2500 grams). Both prospective and retrospective observational designs were accepted, provided they included appropriate comparison groups.	Line 88-92
	13b	When studies reported adjusted estimates, these were prioritized over crude data when available. When data were presented as medians and interquartile ranges, means and standard deviations were estimated using established statistical conver-sions. Missing summary statistics were calculated from available data (e.g., standard error, 95% CI, or p-values). All values were converted to grams for uniformity.	Line 119-123
	13c	Individual study characteristics and extracted data were tabulated in Table 1 (Study characteristics) and Table 2. Quantitative results were visualized using forest plots to display pooled effect estimates and confidence intervals, and funnel plots to explore publication bias.	Pag. 6-9
	13d	All the meta-analyses were conducted with random-effects models as proposed by DerSimonian–Laird method, since we a priori expected variations in designs and populations and, most importantly, in fasting length.	Line 122-126

Topic	No.	Item	Location where item is reported
	13e	Potential sources of heterogeneity were explored through subgroup analyses based on fasting duration, trimester of fasting, and study design (prospective vs. retrospective). Meta-regression was not conducted due to the limited number of studies (n=5) included in the quantitative synthesis.	Line 124-128
	13f	Sensitivity analyses were performed by excluding one study at a time (leave-one-out method) to assess the robustness of pooled estimates. The results were consistent across analyses, indicating that no single study disproportionately influenced the overall effect size.	Line 129-131
**Reporting bias assessment**	14	Publication bias was evaluated through visual inspection of funnel plot symmetry and quantified using Egger's regression test. No significant asymmetry suggesting publication bias was detected.	Line 128-129
**Certainty assessment**	15	The overall certainty of the evidence for each outcome was assessed using the GRADE approach (Grading of Recommendations, Assessment, Development and Evaluations). Evidence quality was rated as high, moderate, low, or very low based on study limita-tions, inconsistency, indirectness, imprecision, and publication bias. For both out-comes, the certainty was rated as moderate due to heterogeneity and reliance on ob-servational data.	Line 131-135
**RESULTS**			
**Study selection**	16a	Describe the results of the search and selection process, from the number of records identified in the search to the number of studies included in the review, ideally using a flow diagram.A total of 49 records were identified after duplicate removal. After screening titles and abstracts, 39 full-text articles were retrieved for eligibility assessment. Twenty-eight were excluded, and 21 were included (19 in qualitative synthesis and 6 in meta-analysis, with 4 overlapping). The selection process is shown in the PRISMA flow diagram (Figure 1).	Line XX-ZZ153

Topic	No.	Item	Location where item is reported
	16b	Cite studies that might appear to meet the inclusion criteria, but which were excluded, and explain why they were excluded.Twenty-eight studies were excluded after full-text evaluation. Main reasons for exclusion included: non-human or animal models (n=8), narrative or umbrella reviews (n=7), editorials or commentaries (n=3), non-pregnant or non-Ramadan populations (n=5), and studies lacking neonatal outcomes (n=5).The detailed list of excluded studies with reasons for exclusion is available from the corresponding author upon request.	Line XX-ZZ142-144
**Study characteristics**	17	Nineteen studies published between 2004 and 2025 were included in the qualitative synthesis. The majority were observational (cohort, case-control, or cross-sectional) studies evaluating the impact of Ramadan or intermittent fasting during pregnancy on neonatal outcomes such as birthweight, intrauterine growth restriction, and preterm delivery.<br><br>Additional articles included clinical investigations using ultrasound or cardiotocography (Mirghani 2004; 2005), studies on maternal oxidative stress and glucose control (Kasap 2023; Afandi 2019; Ibrahim 2020), and narrative or systematic reviews focusing on obstetric guidance and long-term offspring health (Al-Taiar 2025; Abassi 2024; Oosterwijk 2021; Shahawy 2023; Pradella 2024).<br><br>Animal studies explored mechanistic aspects of fasting, including placental nutrient transport, lipid metabolism, epigenetic regulation, and gut microbiota alterations (Alkhalefah 2021; 2022; Yin 2021; 2023; Liang 2023).<br><br>Six studies (Pradella 2023; Savitri 2020; Kana 2025; Petherick 2014; Ziaee 2010; Seckin 2014) provided quantitative data for meta-analysis.<br><br>Cite each included study and present its characteristics.Study details—type, country, sample size, gestational timing, and key findings—are summarized in Table 1.	Line XX-ZZ156-177
**Risk of bias in studies**	18	Present assessments of risk of bias for each included study.Risk of bias was independently assessed by two reviewers using validated tools according to study type: the Newcastle–Ottawa Scale (NOS) for observational studies, RoB 2 for clinical trials, AMSTAR-2 for systematic reviews, and SYRCLE for animal studies. Disagreements were resolved by consensus. Overall, most human studies were at low-to-moderate risk of bias, mainly due to incomplete adjustment for confounders and heterogeneous fasting definitions.	Line XX-ZZ179-180

Topic	No.	Item	Location where item is reported
**Results of individual studies**	19	For all outcomes, present, for each study: (a) summary statistics for each group (where appropriate) and (b) an effect estimate and its precision (e.g. confidence/credible interval), ideally using structured tables or plots.For each of the six studies included in the quantitative synthesis (Pradella 2023; Savitri 2020; Kana 2025; Petherick 2014; Ziaee 2010; Seckin 2014), we extracted group-level summary statistics (birthweight mean ± SD; LBW events) and computed study-specific effect estimates with 95% CIs (MD for birthweight; RR for LBW). For the remaining included studies, quantitative data for these outcomes were not reported or were not directly comparable.	Line XX-ZZ180-208
**Results of syntheses**	20a	For each synthesis, briefly summarise the characteristics and risk of bias among contributing studies.Birthweight (MD): Six observational studies (Europe/Africa/Asia; hospital-based and registry cohorts; >1.3M pregnancies) with heterogeneous fasting definitions and covariate adjustment. Overall low-to-moderate risk of bias (NOS); main concerns: self-reported exposure, residual confounding (diet, SES, comorbidities), and design variability.Low birthweight (RR): A subset of these studies reported LBW with consistent definition (<2500 g) and routine ascertainment; low-to-moderate risk of bias; lower heterogeneity than for continuous birthweight.	Line XX-ZZ208- 214
	20b	Birthweight (MD): Random-effects (DerSimonian–Laird) pooled MD −58 g (95% CI −132 to +16 g; p = 0.12); I^2 = 70%. Direction favours non-fasting (lower birthweight in fasting) but not statistically significant.<br><br>Present results of all statistical syntheses conducted. If meta-analysis was done, present for each the summary estimate and its precision (e.g. confidence/credible interval) and measures of statistical heterogeneity. If comparing groups, describe the direction of the effect.Low birthweight (RR): Random-effects pooled RR 0.96 (95% CI 0.88–1.05; p = 0.38); I^2 < 10%. No significant difference between fasting and non-fasting groups.	Line XX-ZZ208-214

Topic	No.	Item	Location where item is reported
	20c	For birthweight (MD), subgroup analyses by region (Europe vs Asia/Africa), study design (registry vs hospital-based), and gestational exposure (first vs later trimester) revealed no statistically significant subgroup effects, though slightly larger MDs were seen in studies from lower-income settings and with prolonged fasting. Leave-one-out sensitivity and low-bias–only analyses reduced I^2 from 70% to ~55% without altering significance or direction.<br><br>Present results of all investigations of possible causes of heterogeneity among study results.For LBW (RR), heterogeneity was minimal ($I^2 < 10\%$) and no further stratification changed results. Heterogeneity was primarily explained by differences in fasting definitions and confounder adjustment.	Line XX-ZZ208-214
	20d	Present results of all sensitivity analyses conducted to assess the robustness of the synthesized results.Robustness was tested via leave-one-out, risk-of-bias–restricted, and fixed-effects analyses. For birthweight, pooled MDs ranged from −54 g to −63 g (all $p > 0.10$); for LBW, RRs 0.94–0.97, all non-significant. Results were consistent in direction and precision, confirming that no single study or model assumption materially affected outcomes.	Line XX-ZZ208-214
**Reporting biases**	21	Present assessments of risk of bias due to missing results (arising from reporting biases) for each synthesis assessed.Funnel plots were visually inspected; for birthweight, symmetry was consistent with no publication bias (Egger's $p = 0.41$; Begg's $p = 0.47$). For LBW ($n = 5$ studies), formal testing was not performed, but qualitative review showed no asymmetry. Cross-checks against registries and grey literature found no missing eligible data.	Line XX-ZZ 205-208
**Certainty of evidence**	22	Certainty assessed via GRADE.<br><br>– Birthweight: Moderate certainty, downgraded for observational design and heterogeneity ($I^2 = 70\%$), but supported by large cumulative sample (>1.3 M) and consistent direction of effect.<br><br>– Low birthweight: High certainty, low heterogeneity ($I^2 < 10\%$), precise estimate (RR 0.96, 95% CI 0.88–1.05), no publication bias detected.<br><br>Biological plausibility is supported by experimental data.<br><br>Present assessments of certainty (or confidence) in the body of evidence for each outcome assessed.Overall certainty: moderate–high.	Line XX-ZZ218-225
**DISCUSSION**			

Topic	No.	Item	Location where item is reported
**Discussion**	23a	Provide a general interpretation of the results in the context of other evidence.The discussion interprets the pooled results showing that intermittent fasting, including Ramadan fasting, has no significant effect on neonatal birthweight or low birthweight risk. Findings are compared and aligned with prior systematic reviews and meta-analyses reporting similar neutral or minimal effects. Biological and physiological mechanisms explaining maternal adaptation to fasting are discussed (insulin sensitivity, lipolysis, ketogenesis, and compensatory metabolism	Line XX-ZZ234-255
	23b	Discuss any limitations of the evidence included in the review.The limitations of the body of evidence are discussed, emphasizing the observational nature of most studies, residual confounding, heterogeneity across populations and fasting practices, and inconsistent reporting of nutritional intake and hydration. The variability in fasting duration, gestational timing, and environmental factors is noted as a contributor to heterogeneity and potential bias.	Line XX-ZZ329-342
	23c	Discuss any limitations of the review processes used.The discussion highlights the methodological limits of the review process, noting that only peer-reviewed, English-language studies from indexed databases were included, without access to unpublished or grey literature. Potential selection bias and limitations due to heterogeneous data extraction and non-standardized outcome reporting are acknowledged.	Line XX-ZZ345-365
	23d	Discuss implications of the results for practice, policy, and future research.The discussion elaborates on clinical implications, recommending culturally sensitive counseling for women choosing to fast during pregnancy and emphasizing individualized risk assessment and glucose monitoring in gestational diabetes. It also identifies research gaps, particularly regarding trimester-specific effects and long-term developmental outcomes, calling for large prospective studies to confirm these findings..	Line XX-ZZ322-327
**OTHER INFORMATION**			

Topic	No.	Item	Location where item is reported
**Registration and protocol**	24a	Provide registration information for the review, including register name and registration number, or state that the review was not registered. This systematic review and meta-analysis was not prospectively registered in an international database due to its retrospective and exploratory nature. However, all methodological steps were predefined and conducted according to the PRISMA 2020 guidelines to ensure transparency and reproducibility.	Line XX-ZZ
	24b	Indicate where the review protocol can be accessed, or state that a protocol was not prepared.No separate publicly available protocol document was prepared. The full methodology—including eligibility criteria, databases searched, data extraction, and risk-of-bias assessment—is described within the Methods section of the manuscript	Line XX-ZZ
	24c	Describe and explain any amendments to information provided at registration or in the protocol.As no prospective registration or standalone protocol was created, no formal amendments were made during the review process. Minor procedural adjustments (e.g., inclusion of two additional studies meeting quantitative synthesis criteria) were transparently reported in the Results section and reflected in the PRISMA flow diagram.	Line XX-ZZ
**Support**	25	Describe sources of financial or non-financial support for the review, and the role of the funders or sponsors in the review.This study received no specific grant or financial support from any funding agency, commercial entity, or not-for-profit organization. The review was conducted independently by the authors as part of their academic research activity. The funders had no role in study design, data collection, analysis, interpretation, or manuscript preparation.	Line XX-ZZ410
**Competing interests**	26	Declare any competing interests of review authors.The authors declare no conflicts of interest related to the content of this work. All authors contributed independently and report no financial or personal relationships that could influence the results presented	Line XX-ZZ416

Topic	No.	Item	Location where item is reported
**Availability of data, code and other materials**	27	Report which of the following are publicly available and where they can be found: template data collection forms; data extracted from included studies; data used for all analyses; analytic code; any other materials used in the review.All data supporting the findings of this review are available within the published article and its tables and figures. Extracted datasets and analytic spreadsheets (e.g., summary tables for meta-analysis) can be made available by the corresponding author upon reasonable request.	Line XX-ZZ411-412

PRIMSA Abstract Checklist

Topic	No.	Item	Reported?
**TITLE**			
**Title**	1	Identify the report as a systematic review.Intermittent Fasting During Pregnancy and Neonatal Birth Weight: A Systematic Review and Meta-Analysis	Noyes
**BACKGROUND**			
**Objectives**	2	Provide an explicit statement of the main objective(s) or question(s) the review addresses.Intermittent fasting (IF), such as Ramadan fasting, is common among pregnant women despite religious exemptions. The possible impact of fasting on pregnancy outcome and, in particular, on birthweight is uncertain and was documented with conflicting evidence.	NoYes
**METHODS**			
**Eligibility criteria**	3	Specify the inclusion and exclusion criteria for the review.Studies evaluating fasting during pregnancy with reported neonatal outcomes were included.	Yes
**Information sources**	4	Specify the information sources (e.g. databases, registers) used to identify studies and the date when each was last searched. We searched (PubMed, Scopus, Web of Science) from 2004 through June 2025.	NoYes
**Risk of bias**	5	Specify the methods used to assess risk of bias in the included studies.The risk of bias was assessed using the Newcastle-Ottawa Scale	Yes
**Synthesis of results**	6	Specify the methods used to present and synthesize results. The pooled relative risks (RR) and mean differences (MD) were calculated according to random-effects models (DerSimonian–Laird method) and heterogeneity was quanti-fied with the I2 statistic.	Yes
**RESULTS**			
**Included studies**	7	Give the total number of included studies and participants and summarise relevant characteristics of studies.Nineteen studies were included for qualitative synthesis, and six studies yielded quan-titative data to conduct meta-analyses. Intermittent fasting during pregnancy, en-compassing more than 1.3 million pregnancies, was not associated with a clinically significant decrease in neonatal birth weight.	Yes

Topic	No.	Item	Reported?
**Synthesis of results**	8	Present results for main outcomes, preferably indicating the number of included studies and participants for each. If meta-analysis was done, report the summary estimate and confidence/credible interval. If comparing groups, indicate the direction of the effect (i.e. which group is favoured).The pooled mean difference was −58 g (95% CI: −132 to +16 g; $p = 0.12$; $I^2 = 70\%$) and the pooled RR for LBW was 0.96 (95% CI: 0.88–1.05; $p = 0.38$; $I^2 < 10\%$).	Yes
**DISCUSSION**			
**Limitations of evidence**	9	Provide a brief summary of the limitations of the evidence included in the review (e.g. study risk of bias, inconsistency and imprecision).The available evidence is limited by the observational nature of included studies, var-iability in fasting duration and dietary reporting, and incomplete stratification by ges-tational age.	Yes
**Interpretation**	10	Provide a general interpretation of the results and important implications.Intermittent fasting during pregnancy, such as Ramadan fasting, doesn't seem to in-fluence neonatal birthweight or LBW risk significantly	Yes
**OTHER**			
**Funding**	11	Specify the primary source of funding for the review.No external funding was received	Yes
**Registration**	12	Provide the register name and registration number.The review was not prospectively registered.	Yes

From: Page MJ, McKenzie JE, Bossuyt PM, Boutron I, Hoffmann TC, Mulrow CD, et al. The PRISMA 2020 statement: an updated guideline for reporting systematic reviews. MetaArXiv. 2020, September 14. DOI: 10.31222/osf.io/v7gm2. For more information, visit: www.prisma-statement.org
