# Supplementary material for: Intermittent Fasting During Pregnancy and Neonatal Birth Weight: A Systematic Review and Meta-Analysis
Source: Nutrients. 2025 Nov 13;17(22):3546. doi: 10.3390/nu17223546 (PMC12655342; doi:10.3390/nu17223546)
Supplement: Supplementary file 1 [file nutrients-17-03546-s001.zip › Table 1 and Table 2 supplementary.pdf]

Author (year)	Type of study	Country / context	Sample Size	Trimester	Focus/ theme	Key findings	
Al-Taiar (2025)	Umbrella review	Multicenter	>100.000	All	Neonatal outcomes	Mixed effects on birth weight and preterm birth	
Abassi (2024)	Systematic narrative review	Multicenter	n/a	All	Long-term outcomes	Possible effect on metabolic health and stature	entry 2 data data 1
Kasap (2023)	Observational study	Turkey	92	2nd	Maternal oxidative stress	↓ (TAC) and ↑ (MDA) with fasting >15 days	
Shahawy (2023)	Clinical review	USA	n/a	All	Obstetric counseling	Individualized approach recommended	
Kana (2025)	Observational study	Nigeria	400	2nd–3nd	Nutrition and neonates	Birth weight influenced by post-fasting diet quality	
Gur (2015)	Observational study	Turkey	65	All	Maternal metabolic parameters	Non-significant metabolic variations	
Pradella (2023)	Cross-sectional study	Italy	240	1st	Neonatal outcomes	Slight reduction in birth weight	
Savitri (2020)	Cohort study	Netherlands	1.300	1st	IUGR e Ramadan	↑ risk of IUGR in vulnerable subgroups	
Oosterwijk (2021)	Systematic review	Multinational	n/a	Various	Neonatal outcomes	Modest effects, dependent on fasting duration	
Petherick (2014)	Cohort study	United Kingdom	n.d.	All	Preterm birth	No significant correlation	
Mirghani (2004)	Clinical study (ultrasound)	Sudan	38	2 nd	Fetal movements	↓ Fetal breathing movements	
Mirghani (2005)	Clinical study (CTG – Cardiotocography)	Sudan	44	2nd	Fetal well-being	↓ Fetal heart rate variability	
van Ewijk (2013)	Longitudinal study	Indonesia	>20.000	1st	Adult outcomes	↓ Height and school performance	
Pradella (2024)	Meta-analysis	Multinational	n/a	All	Long-term outcomes	Associations with growth and metabolism	
Afandi (2019)	Observational study	EAU	27	3nd	Gestational diabetes mellitus (GDM) and continuous glucose monitoring (CGM)	Better glycemic control with CGM	
Ibrahim (2020)	Practical study	EAU	n/a	All	Diabetes e Ramadan	Recommended use of CGM	
Alkhalefah (2021)	Experimental animal study	Saudi Arabia	mice	Mid-to-late	Placenta	↓ Placental amino acid transport	
Alkhalefah (2022)	Animal study	Saudi Arabia	mice	All	Placental metabolism	Lipid alterations and fetal growth	

Yin (2023)	Animal study	China	mice	Late	Hepatic metabolism	↓ mTORC1 and liver function in the offspring
Yin (2021)	Epigenetic study	China	mice	Pre-gestation	Epigenetics	Altered fetal DNA methylation
Liang (2023)	Animal study	China	mice	All	Gut microbiota	Dysbiosis and intestinal inflammation

**Author (year)**	**Country**	**N (fasting)**	**N (non-fasting)**	**Medium weight(g) fasting ± SD**	**Medium weight (g) non-fasting ± SD**	**LBW <2500 g fasting (n/N)**	**LBW <2500 g non-fasting (n/N)**	**Trimester**	**Note**
Pradella (2023)	Italy	98	207	3307 ± 511	3373 ± 540	4/98 (4%)	10/207 (5%)	1° :117 , 2° : 92 , 3° : 116	cross-sectional study, BMI-adjusted analysis
Savitri (2020)	Netherlands	116,01	1,219,606	3420.3±582.7	3464.3±599.7	6,491 / 116,010	72,829 / 1,219,606	Any trimester	dichotomous LBW data reliable for meta-analysis; very small mean weight differences
Kana (2025)	Nigeria	1158	212	3005.96 ± 472.78	3206.74 ± 514.82	n.d.	n.d.	2°–3°	Significantly lower mean weight in fasting; lbw data non reported
Ziaee et al., 2010	Iran	123	66	3043 ± 577	3010 ± 500	–	–	1°	Combined fastiing; no significant difference between fasting and non- fasting;
Seckin et al., 2014	Turchia	82	87	3089 ± 300	3450 ± 352	–	–	≥20 fasting days	Fasting ≥20 days; no significant difference in birth weight, gestational age, or delivery type; reduced AFI in the fasting group
Pheterick et al., 2014	UK	128	172	3219.3 ± 534.4	3133 ± 467.4	8/128 (6.3%)	14/172 (8.1%)	1°: 42, 2°: 74, 3°: 11	Prospective study of Muslim women; fasting not associated with significant differences in birth weight, lbw, or preterm birth
