# Supplementary material for: Intermittent Fasting During Pregnancy and Neonatal Birth Weight: A Systematic Review and Meta-Analysis
Source: Nutrients. 2025 Nov 13;17(22):3546. doi: 10.3390/nu17223546 (PMC12655342; doi:10.3390/nu17223546)
Supplement: Supplementary file 1 [file nutrients-17-03546-s001.zip › Table S1. risk of bias supplementary.pdf]

Table S1. Risk of bias assessment of included studies

Author (year)	Study type	Assessment tool	Risk of bias	Main limitation
**Al-Taiar (2025)**	Umbrella review	AMSTAR-2	Moderate	Overlap of included studies, lack of sensitivity analysis
**Abassi (2024)**	Systematic narrative review	AMSTAR-2	Moderate	Heterogeneous study designs, incomplete reporting of search strategy
**Kasap (2023)**	Observational study	NOS	Moderate	Small sample size, lack of randomization and adjustment for confounders
**Shahawy (2023)**	Clinical review	Not applicable	–	Narrative format, expert opinion only
**Kana (2025)**	Observational study	NOS	Low	Limited dietary data but good methodological clarity
**Gur (2015)**	Observational study	NOS	Moderate	Small sample, single-center, lack of control for caloric intake
**Pradella (2023)**	Cross-sectional study	NOS	Low	Potential recall bias in fasting exposure data
**Savitri (2020)**	Cohort study	NOS	Low	Missing adjustment for socioeconomic variables
**Oosterwijk (2021)**	Systematic review	AMSTAR-2	Moderate	Moderate heterogeneity across included studies
**Petherick (2014)**	Cohort study	NOS	Low	Potential under-reporting of fasting adherence
**Mirghani (2004)**	Clinical (ultrasound)	RoB 2 (adapted)	Moderate	Lack of blinding, small sample
**Mirghani (2005)**	Clinical (CTG)	RoB 2 (adapted)	Moderate	No control group, small cohort
**van Ewijk (2013)**	Longitudinal study	NOS	Low	Exposure data based on calendar approximation
**Pradella (2024)**	Meta-analysis	AMSTAR-2	Low	Limited subgroup analyses
**Afandi (2019)**	Observational study (GDM)	NOS	Moderate	Small sample, limited duration of follow-up

Author (year)	Study type	Assessment tool	Risk of bias	Main limitation
**Ibrahim (2020)**	Practical report	Not scored	–	Non-systematic, descriptive
**Alkhalefah (2021)**	Animal study	SYRCLE	Moderate	Lack of blinding and random allocation details
**Alkhalefah (2022)**	Animal study	SYRCLE	Moderate	Missing outcome assessor blinding
**Yin (2023)**	Animal study	SYRCLE	Moderate	Incomplete randomization details
**Yin (2021)**	Animal study	SYRCLE	Moderate	Epigenetic outcomes with small litter numbers
**Liang (2023)**	Animal study	SYRCLE	Moderate	Lack of control for maternal weight and feed intake

Overall summary:

• **Humans (n=14)** → Low-moderate risk of bias, mainly due to variability in exposure and small sample sizes.

• **Reviews (n=3)** → Moderate methodological quality, limited by the heterogeneity of the included studies.

• **Animals (n=5)** → Moderate bias, due to lack of detail on randomisation and blinding.
