# Supplementary material for: Intermittent Fasting During Pregnancy and Neonatal Birth Weight: A Systematic Review and Meta-Analysis
Source: Nutrients. 2025 Nov 13;17(22):3546. doi: 10.3390/nu17223546 (PMC12655342; doi:10.3390/nu17223546)
Supplement: Supplementary file 1 [file nutrients-17-03546-s001.zip › Table S2. Excluded studies supplementary.pdf]

Final Numerical Verification

Total articles identified after deduplication: 49

Included in qualitative review: 19

Included in meta-analysis: 6 (of which 4 already in the 19, + 2 new → Ziaee 2010 and Seckin 2014)

Total articles assessed in full text: 21

Excluded after full-text evaluation: 28

Overall total: 49 = 28 excluded + 21 included (19 qualitative + 2 quantitative only)

Table S2 – Studies excluded after full-text evaluation (n = 28)

No.	Author (Year)	Full Title	Reason for Exclusion
1	Darmaun D. (2021)	Maternal intermittent fasting during pregnancy: a translational research challenge for an important clinical scenario. Clin Sci (Lond).	Editorial/commentary, not an original study.
2	Alkhalefah A. et al. (2022)	Impact of maternal intermittent fasting during pregnancy on cardiovascular, metabolic and renal function in adult rat offspring. PLoS One.	Animal model study.
3	Ibrahim M. et al. (2020)	Recommendations for management of diabetes during Ramadan: update 2020. BMJ Open Diabetes Res Care.	Clinical guidelines, no original data on neonatal outcomes.
4	Alkhalefah A. et al. (2021)	Maternal intermittent fasting during pregnancy induces fetal growth restriction… Clin Sci (Lond).	Animal model study.

5	Jiang X. et al. (2022)	Overexpression of Pregnancy Zone Protein in Fat Antagonizes Diet-Induced Obesity Under an Intermittent Fasting Regime. Front Physiol.	Not related to pregnancy or neonatal outcomes.
6	Terada M. (1970)	Effect of intermittent fasting before pregnancy upon maternal fasting as a teratogen in mice. J Nutr.	Pre-clinical study, not on pregnant women.
7	Yin W. et al. (2023)	Maternal intermittent fasting deteriorates offspring metabolism via suppression of hepatic mTORC1 signaling. FASEB J.	Animal study.
8	Wang Y. et al. (2024)	Treatment of mice with maternal intermittent fasting to improve fertilization rate and reproduction. Zygote.	Animal model study.
9	Liang Y. et al. (2023)	Maternal intermittent fasting in mice disrupts the intestinal barrier leading to metabolic disorder in adult offspring. Commun Biol.	Animal study.
10	Yin W. et al. (2021)	Maternal intermittent fasting before mating alters hepatic DNA methylation in offspring. Epigenomics.	Animal study.
11	Zhu S. et al. (2021)	Meal Timing and Glycemic Control during Pregnancy—Is There a Link? Nutrients.	Does not address intermittent fasting or Ramadan.
12	Velissariou M. et al. (2025)	The impact of intermittent fasting on fertility: A focus on PCOS and reproductive outcomes in women. Metabol Open.	Not related to pregnant women.

13	Obaideen K. et al. (2022)	Seven decades of Ramadan intermittent fasting research: Bibliometrics analysis. Diabetes Metab Syndr.	Bibliometric analysis, not a clinical study.
14	Ali A.M., Kunugi H. (2020)	Intermittent Fasting, Dietary Modifications, and Exercise for the Control of Gestational Diabetes and Maternal Mood Dysregulation: A Review and a Case Report. IJERPH.	Narrative review, no primary data.
15	Chouli M. et al. (2025)	An updated review of popular dietary patterns during pregnancy and lactation: Trends, benefits, and challenges. Metabol Open.	General narrative review.
16	Flanagan E.W. et al. (2022)	Assessment of Eating Behaviors and Perceptions of Time-Restricted Eating During Pregnancy. J Nutr.	Descriptive study, no neonatal outcomes.
17	SANDS R.X. (1964)	Intermittent modified total-fasting in the treatment of obstetric obesity. Am J Obstet Gynecol.	Therapeutic non-religious intervention, not comparable.
18	Gray K.L. et al. (2021)	Intermittent energy restriction on weight loss and diabetes risk markers in women with prior gestational diabetes. Am J Clin Nutr.	Study on non-pregnant women.
19	Bucknor M.C. et al. (2024)	High fat diet consumption and social instability stress impair stress adaptation and maternal care in C57Bl/6 dams. Psychoneuroendocrinology.	Animal study.

20	Fikadu T. et al. (2024)	Determinants of breakfast skipping among pregnant women from South Ethiopia. Sci Rep.	Not related to intermittent fasting or Ramadan.
21	Kamel M. et al. (2012)	Effets de la restriction alimentaire appliquée à des rates adultes sur la croissance osseuse… Pathol Biol (Paris).	Animal study.
22	Guilfoyle M.M. (2024)	Ramadan fasting in the third trimester of pregnancy and postpartum colostrum cortisol concentrations in Morocco. Am J Hum Biol.	Hormonal outcomes, not neonatal.
23	Mao L. et al. (2024)	Effects of Intermittent Fasting on Female Reproductive Function: A Review of Animal and Human Studies. Curr Nutr Rep.	Narrative review, no original data.
24	AbuShihab K. et al. (2024)	Reflection on Ramadan Fasting Research Related to SDG 3 (Good Health and Well-Being). J Relig Health.	Bibliometric analysis.
25	Phalle A., Gokhale D. (2025)	Maternal and fetal outcomes in gestational diabetes mellitus: a narrative review of dietary interventions. Front Glob Womens Health.	Narrative review, not original study.
26	Susser E., Ananth C.V. (2013)	Invited commentary: is prenatal fasting during Ramadan related to adult health outcomes? Am J Epidemiol.	Editorial/commentary, no original data.
27	Mohany M. et al. (2018)	A new model for fetal programming: maternal	Animal study.

		Ramadan-type fasting programs nephrogenesis. J Dev Orig Health Dis.	
28	Al-Taiar A. et al. (2025)	Impacts of Ramadan fasting during pregnancy on pregnancy and birth outcomes: An umbrella review. Int J Gynaecol Obstet.	Umbrella review, no primary data.

PRISMA Summary Consistency

Category	No. of Studies
Total records identified	49
Full-text articles assessed	39
Excluded after full-text	28
Included in qualitative review	19
Included in meta-analysis	6
